# Autism-like phenotype across the lifespan of *Shank3B*-mutant mice of both sexes

**DOI:** 10.1186/s11689-025-09635-3

**Published:** 2025-08-02

**Authors:** Jakub Szabó, Johan Filo, Rebeka Démuthová, Emese Renczés, Veronika Borbélyová, Daniela Ostatníková, Peter Celec

**Affiliations:** 1 Institute of Molecular Biomedicine, Faculty of Medicine, Comenius University, Bratislava, Slovakia; 2 Institute of Medical Physics and Biophysics, Faculty of Medicine, Comenius University, Bratislava, Slovakia; 3 Institute of Physiology, Faculty of Medicine, Comenius University, Bratislava, Slovakia; 4 Institute of Pathophysiology, Faculty of Medicine, Comenius University, Bratislava, Slovakia

**Keywords:** Animal model, Phelan-McDermid syndrome, Phenotyping, Symptom development

## Abstract

**Background:**

High heritability (80–90%) of the autism spectrum disorder (ASD) and sex-biased incidence (3–4 times more boys than girls) suggest the roles of genetic predisposition and sex in the etiopathogenesis of the disorder. As ASD is commonly diagnosed in early childhood, most of the research is focused on children, yet animal research predominantly uses adult-aged animals. The effect of aging on the core and secondary ASD symptomatology is understudied, both in patients and animal models of ASD.

**Methods:**

To investigate the effect of aging on sociability, repetitive behavior, exploration, locomotor activity, anxiety-like behavior, and object-avoidance behavior, behavioral phenotyping was conducted in *Shank3B*^*−/−*^ (*n* = 67) and C57BL/6J wild-type (WT, *n* = 68) mice of both sexes (female *n* = 70, male *n* = 65) in adolescence (1–2 months of age, *n* = 42), adulthood (3–6 months of age, *n* = 40), and old age (12–18 months of age, *n* = 53).

**Results:**

Social deficits were observed only in old *Shank3B*^*−/−*^ males. Anxiety-like behavior peaked in adulthood with *Shank3B*^*−/−*^ mice roughly 20% more anxious than controls. Repetitive grooming and object-induced avoidance behavior were twice more prevalent in *Shank3B*^*−/−*^ mice consistently across the lifespan. Hypoactivity (20% less distance moved) and reduced exploration (30% less rearing behavior) were recorded in *Shank3B*^*−/−*^ mice and were more prevalent in female animals (30% less rearing behavior). Data were analyzed using the Three-way ANOVA (genotype, sex, age), followed by a posthoc Bonferroni correction to compare respective subgroups.

**Conclusions:**

Present study shows that aging affects ASD-like phenotype in the *Shank3B*-mutant mouse model, even though the effect size seems to be small. The mechanisms underlying these partially sex-specific effects should be the subject of further research with potential translational implications.

## Introduction

Autism spectrum disorder (ASD) is an umbrella term for a group of neurodevelopmental disorders. Despite the united diagnostic classification, ASD is highly heterogeneous both in clinical manifestation and in etiopathogenesis. Although its symptomatology is variable, it is generally defined by two major symptoms – deficient sociability and repetitive behavior [1]. Broader ASD symptoms include locomotor and sensory deficits, anxiety, and limited interest. A distinct role of genetic risk factors, male sex, as well as sex steroid hormones were previously shown to be implicated in the etiopathogenesis of ASD [6, 7, 33, 53].

Whole-genome studies identified around 100 genes associated with ASD [55], many of which encode synaptic proteins [49]. One of them, *SH3 and multiple ankyrin repeat domains 3* (SHANK3) encodes an eponymous excitatory postsynaptic protein that plays a crucial role in regulating postsynaptic receptors as well as signaling molecules [41]. Its haploinsufficiency causes Phelan-McDermid syndrome, a rare neurodevelopmental condition characterized by ASD-like symptoms [48]. Although only about 1% of ASD patients carry the SHANK3 mutation, the gene is among the best-characterized genes implicated in ASD [16], and its disruption is hypothesized to be one of the main causes of the associated neurobehavioral deficits [46]. Indeed, *Shank3B*-mutant mice exhibit a variety of ASD-like phenotypes, such as extensive self-injurious stereotypic behavior, abnormal social behavior, anxious manifestations, impaired communication, learning, and memory [11, 68, 69].

Positive male sex association with ASD prevalence and severity of its symptoms reported in human patients has been confirmed in various animal models as well [18, 24, 27, 52, 56]. Using prenatal valproic acid to induce ASD-like symptoms in both mouse and rat models resulted in social deficits in male, but not female animals [14, 22, 29, 56]. Male-specific social deficits, but also anxiety-like behavior, were recorded in mouse ASD models induced by polycytidylic acid [24, 25]. Social deficits and repetitive behavior were reported to be more pronounced in male mice carrying *Shank3B* mutation following sleep disruption as well [35].

Even though ASD is a lifelong condition [1], due to the very early onset of the disorder, the vast majority of human ASD research is focused on early childhood and school-age years [50]. On the other hand, animal experiments predominantly utilize adult-aged animals [61]. This discrepancy limits the translational potential of animal ASD research. In the current study, we aimed to elucidate whether the behavioral phenotype of the *Shank3B*^*−/−*^ genetic mouse model of ASD varies across the lifespan and if these behavioral manifestations are sex-specific.

## Material & methods

### Animals

From the breeding of heterozygous pairs of *Shank3B*^*+/−*^ mice (obtained from the Jackson Laboratory - JAX Stock No. 017688), *Shank3B*^*+/+*^ (WT; *n* = 37 female, 31 male) and *Shank3B*^*−/−*^ (KO; *n* = 33 female, 34 male) mice were obtained and used in the experiment. At weaning, pups were genotyped, and group-housed (4–5 per cage) with regard to sex and age. Animals were kept in a controlled environment of 24 ± 2 degrees Celsius and 55 ± 10% humidity with *ad libitum* access to food and water on a 12-hour light/dark cycle (lights on at 0600, lights off at 1800). The experiment was performed in accordance with the *Policies on the Use of Animals and Humans in Neuroscience Research* and the *Animal Research: Reporting of In Vivo Experiments (ARRIVE)* guideline [57].

### Genotyping

To confirm the deficiency of the SHANK3 gene, end-point PCR was utilized. Tail samples (~ 0.2 cm) were obtained and processed using the MyTaq™ Extract-PCR Kit (Bioline) according to the manufacturer. After the extraction, genotyping was conducted using a protocol provided by the manufacturer (Jackson Laboratory - JAX Protocol 28553). Three primers designed to identify the WT and KO alleles were used (P1-Common: GAG ACT GAT CAG CGC AGT TG; P2-WT: TGA CAT AAT CGC TGG CAA AG; P3-KO: GCT ATA CGA AGT TAT GTC GAC TAG G). For the WT allele, a 374-bp band was produced, for the KO allele, a 153-bp band was produced and both bands were produced for the heterozygous allele. The PCR products were visualized on a 1.5% agarose gel stained with the GoodView™ Nucleic Acid Stain (Beijing SBS Genetech Co., Ltd.).

### Behavioral testing

Animals were tested in three separate cohorts at three different stages of life – adolescence (1–2 months of age; WT females *n* = 11, Shank3B^−/−^ *n* = 10, WT males *n* = 10, Shank3B^−/−^ *n* = 11), adulthood (3–6 months of age; WT females *n* = 10, Shank3B^−/−^ *n* = 10, WT males *n* = 10, Shank3B^−/−^ *n* = 10), and old age (12–18 months of age; WT females *n* = 16, Shank3B^−/−^ *n* = 13, WT males *n* = 11, Shank3B^−/−^ *n* = 13). Behavior was assessed using the Three-chamber social interaction test, Open field test, Elevated plus-maze, Light-dark box, and Marble burying assay. All tests were carried out in a dimly lit room (average of 40 lx, if not stated otherwise), with a room temperature of 24 ± 1 degrees Celsius. All animals were habituated to the testing room at least 30 min before each test. The behavior was recorded using a dedicated video recorder and the files were analyzed using the video processing software EthoVision XT 10.0 (Noldus Information Technology, Wageningen, Netherlands).

### Sociability

To assess sociability, the Three-Chamber Social Interaction test (SIT) was utilized [17, 60]. Top-open plastic apparatus (60 cm x 40 cm x 20 cm) divided into 3 chambers (20 cm x 40 cm x 20) by 2 clear plastic walls was used. Each plastic wall had a retractable doorway allowing access to other chambers upon opening. The experimental mouse was habituated to the apparatus for 5 min. Sex- and age-matched C57BL/6J social-partner mouse was placed in a cylindrical plastic wire cup (10 cm in diameter) in one of the side chambers, alternating per trial. An identical empty plastic wire cup was placed in an opposite chamber. After the habituation period, the experimental mouse was left to freely explore the apparatus for 10 min. The time spent in each chamber, as well as the time spent interacting with the empty wire cup (object) or the wire cup containing the social partner mouse, was measured. Interaction with either was defined as when the experimental mouse was in close proximity (approximately 1 cm) and oriented toward the cups.

### Repetitive behavior, exploration, and locomotor activity

The Open field test was used [14, 40] to record repetitive behavior (grooming), exploration (rearing), and locomotor activity. Mice were tested in the PhenoTyper 4500 cage (45 cm x 45 cm x 55 cm) virtually divided into a peripheral border zone and a central zone (20 cm x 20 cm). Animals were individually placed into the central zone of the arena and left to freely explore for 10 min while being recorded. To assess locomotor activity, the traveled distance was measured. Cumulative time spent grooming and rearing was manually scored to assess repetitive behavior and explorative behavior, respectively.

### Anxiety-like behavior

The Elevated plus-maze test was conducted to assess thigmotactic anxiety-like behavior [12, 42]. The apparatus consisted of two opposite open (50 cm x 10 cm) and two opposite closed arms (50 cm x 10 cm, with 40 cm high walls) extended from the central platform (10 cm x 10 cm) elevated 60 cm above the floor. Open arms were illuminated by a lamp with 100–120 lx. Each mouse was placed on the central platform facing an open arm and left to freely explore the maze for 5 min. The time spent in open arms was assessed as anti-anxiety behavior.

To further assess bright light-driven anxiety-like behavior, the Light-dark box test was conducted [10, 20, 34]. Animals were tested in a plastic box (60 cm x 40 cm x 30 cm) divided into 2 equally sized chambers (30 cm x 40 cm x 30 cm each), connected with an entrance to allow free transition between the chambers. One chamber was top-open and illuminated by a lamp with 400 lx, whereas the other chamber was top-closed and dark (2 lx). Mice were placed into the light side of the apparatus with their backs to the entrance and left to freely explore for 5 min. Time spent in each chamber was recorded, and light-part exploration was considered a measure of anti-anxiety behavior.

### Marble burying

To analyze object-avoidant behavior [32], the Marble burying test was conducted [2, 47] in a plastic box (40 cm x 25 cm x 30 cm) containing 5 cm of sawdust bedding. Twenty transparent glass marbles were arranged in a 4 × 5 grid and placed on top of the bedding. The animals were individually placed in the center of the cage for 20 min of free movement. The marbles that were at least 50% covered in sawdust were considered buried. A lower number of marbles buried indicated an elevated extent of object-avoidance behavior.

### Statistical analyses

Statistical analyses were conducted using IBM SPSS Statistics 23.0 (IBM, Armonk, NY, USA). The Shapiro-Wilk test was used to determine the normality of the distributions, and the Levene Test for Equality of Variances was used to determine the homogeneity of variances in all measured constructs. To analyze the interactive effects of genotype, sex, and age factors, the Three-way Analysis of Variance (ANOVA) was used. To further compare the respective subgroups, One-way ANOVA followed by the posthoc analysis using the Bonferroni correction were used. Data are presented as mean and SEM. The *p-*values of less than 0.05 were considered significant.

## Results

### Sociability

**Fig. 1:**
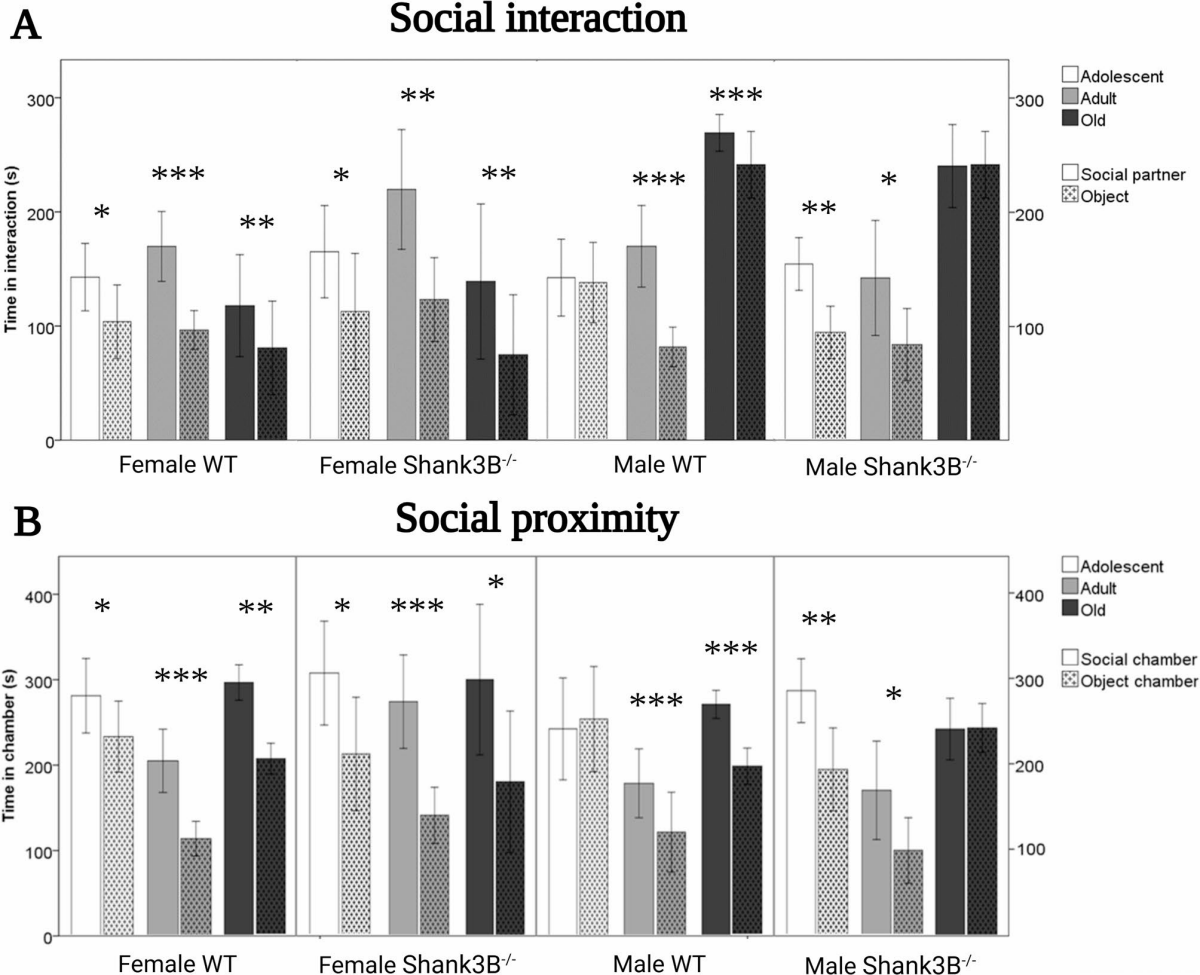
Social interaction assessed by the Three-chamber Social Interaction test (SIT). **A** Preference measured by cumulative time spent interacting with either a social partner or an object. Age effect (adult > adolescent *p* = 0.037, adult > old *p* = 0.038). Effect of age & sex & genotype (no preference in old *Shank3B*^*−/−*^ males). **B** Social proximity assessed by SIT. Preference measured by cumulative time spent in the chamber with either the social partner or an object. Sex effect (female > male, *p* = 0.033). *WT* = wild-type mice; *Shank3B*^*−/−*^=*Shank3B* homozygous knock-out mice. *=*p* < 0.05, **=*p* < 0.01, ***=*p* < 0.001

In Social interaction, the main effect of age was observed to be the preference for an interaction with a social partner (mouse) over an object [F(2, 134) = 4.21, *p* = 0.017], but no main effects of genotype or sex were found. Adult animals spent approximately twice as much time in social interaction (79.1 s. ± 7.9) compared to the adolescent (39.5 s. ± 13.4, *p* = 0.037) and old (41.8 s. ± 9.4, *p* = 0.038, Fig. 1A) mice. Furthermore, an interactive effect of age, genotype, and sex was observed [*F*(2, 134) = 3.203, *p* = 0.044]. Old male *Shank3B*^*−/−*^ mice were the only subgroup that did not exhibit a preference for an interaction with a social partner over an object when compared to the remaining subgroups (Fig. 1A). For time spent in the social chamber, the main effect of sex was recorded [F(1, 134) = 4.39, 90.9 s. ± 15.7], but no main effects of age and genotype. The female mice preferred spending time in the social chamber over the object chamber twice as much (90.9 s. ± 15.7) compared to the males (45.9 s. ± 13.5, *p* = 0.033, Fig. 1B).

### Repetitive behavior

**Fig. 2:**
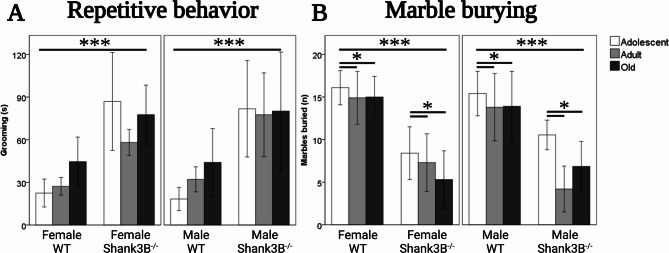
**A** Repetitive behavior assessed in the Open field test measured by cumulative time spent self-grooming. Genotype effect (*Shank3B*^*−/−*^>WT, *p* < 0.001). **B** Object avoidance assessed in the Marble burying test measured by the number of marbles buried. Genotype effect (*Shank3B*^*−/−*^< WT, *p* < 0.001). Age effect (adolescent > adult *p* = 0.03, adolescent > old *p* = 0.05). *WT* = wild-type mice; *Shank3B*^*−/−*^=*Shank3B* homozygous knock-out mice. *=*p* < 0.05, ***=*p* < 0.001

In repetitive self-grooming, the main effect of genotype was observed across all animal groups [*F*(1, 134) = 50.33, *p* < 0.001], but no main effects of age or sex were recorded. The *Shank3B*^*−/−*^ mice exhibited over twice as much grooming (77.2 s. ± 5.6) when compared to their WT controls (32.7 s. ± 2.9, Fig. 2A). No factor interactions were observed.

### Exploration

**Fig. 3:**
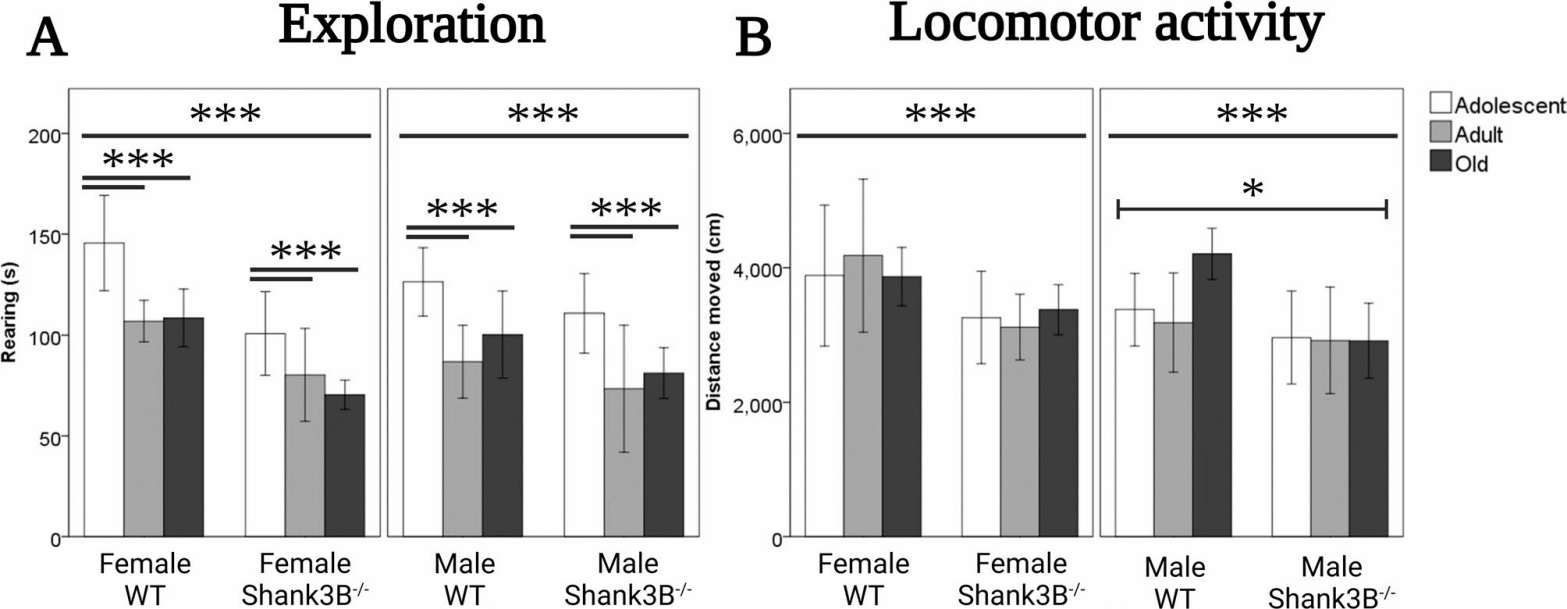
**A** Exploration assessed in the Open field test measured by cumulative time spent rearing. Age effect (adolescent > adult, *p* < 0.001, adolescent > old, *p* < 0.001). Genotype effect (*Shank3B*^*−/−*^< WT, *p* < 0.001). Effect of sex & genotype (female *Shank3B*^*−/−*^< female WT, *p* = 0.037). **B** Locomotor activity assessed in the Open field test by the distance moved. Sex effect (male < female, *p* = 0.045). Genotype effect (*Shank3B*^*−/−*^< WT, *p* < 0.001).). *WT* = wild-type mice; *Shank3B*^*−/−*^=*Shank3B* homozygous knock-out mice. *=*p* < 0.05, ***=*p* < 0.001

In explorative rearing behavior, we observed a main effect of age [*F*(2, 134) = 19.26, *p* < 0.001], and a main effect of genotype [*F*(1, 134) = 29.27, *p* < 0.001], but no main effects of sex. The adolescent mice were exploring their surroundings over 25% more (121.2 s. ± 5.1) compared to adult (86.9 s. ± 5, *p* < 0.001) and old mice (90.7 s. ± 3.8, *p* < 0.001, Fig. 3A). The *Shank3B*^*−/−*^ mice displayed over 30% less rearing behavior (85.6 s. ± 3.8) than their WT controls (112.4 s. ± 3.9, Fig. 1E). Furthermore, an interactive effect of genotype and sex was observed [*F*(1, 134) = 4.46, *p* = 0.037]. Female mice exhibited virtually 30% less time spent exploring the surroundings when carrying the *Shank3B*-deficiency (82.6 s. ± 5.5), compared to their WT counterparts (119.1 s. ± 5.1, Fig. 3A), but no such difference was observed in males (*p* = 0.07).

### Locomotor activity

For the distance traveled over the 10-minute period, the main effect of genotype [*F*(1, 134) = 15.75, *p* < 0.001], and the main effect of sex was observed [*F*(1, 134) = 4.11, *p* = 0.045], but no main effects of age. Compared to WT controls (3783.51 cm ± 123.25), *Shank3B*^*−/−*^ mice demonstrated approximately 20% less movement (3092.29 cm ± 123.3, Fig. 3B). The male mice exhibited roughly 10% less distance moved (3255.16 cm ± 123. 25) compared to females (3629.87 cm ± 131.52, Fig. 3B). No factor interactions were observed.

### Anxiety-like behavior

**Fig. 4:**
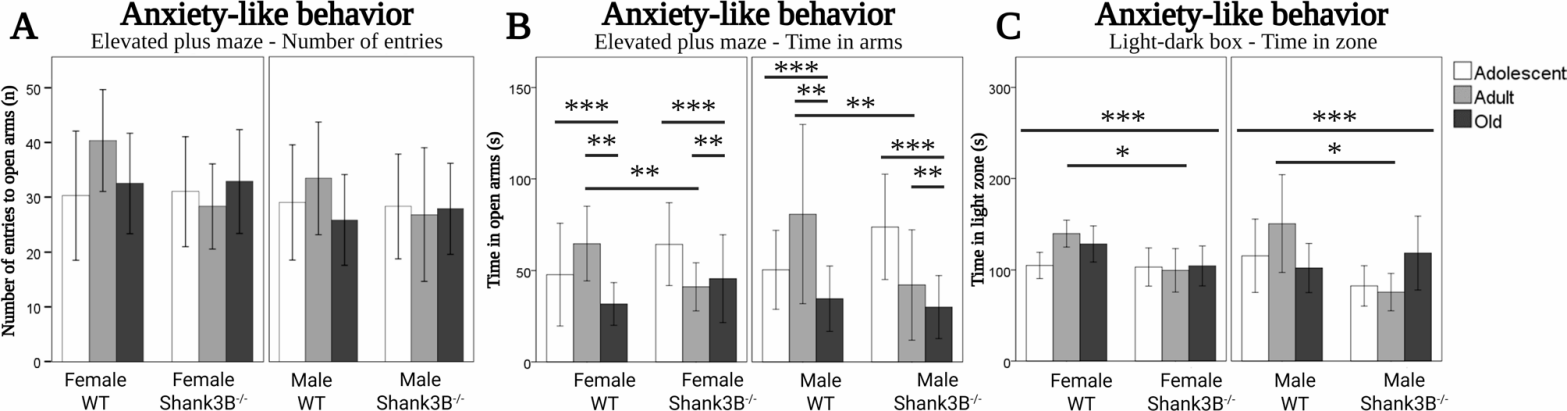
Anxiety-like behavior assessed in the (**A**) Elevated plus maze measured by entries to the open arms, (**B**) cumulative time spent in open arms. Age effect (old < adolescent, *p* < 0.001, old < adult, *p* = 0.006). Effect of age & genotype (adult *Shank3B*^*−/−*^< adult WT, *p* = 0.006). **B** Light-dark box measured by cumulative time spent in the light zone. Genotype effect (*Shank3B*^*−/−*^< WT, *p* < 0.001). Effect of age & genotype (adult *Shank3B*^*−/−*^< adult WT, *p* = 0.012). *WT* = wild-type mice; *Shank3B*^*−/−*^=*Shank3B* homozygous knock-out mice. *=*p* < 0.05, **=*p* < 0.01, ***=*p* < 0.001

No main effects or interactive effects were observed for the number of entries into the open arms of the Elevated plus maze (Fig. 4A). However, for the cumulative time spent in the open arms of the test, there was a main effect of age [*F*(2, 134) = 6.15, *p* = 0.003], but no main effects of genotype, or sex were observed. Old mice spent 40% less time in open arms (35.3 s. ± 4) than the adolescent (59.2 s. ± 5.8, *p* < 0.001) and adult animals (57.2 s. ± 7.1, *p* = 0.006, Fig. 4B). An interactive effect of age and genotype was observed [*F*(2, 134) = 5.28, *p* = 0.006]. The *Shank3B*^*−/−*^ mice explored the open arms approximately half as much in adult age (41.6 ± 8.2) compared to their WT controls (72.8 s. ± 8.2, Fig. 4B), but no similar difference was observed in adolescence (*p* = 0.08) or in old age (*p* = 0.55).

For the time spent in the light zone of the Light-dark box, the main effect of genotype [*F*(1, 134) = 12.22, *p* < 0.001], but no main effects of age or sex were observed. The *Shank3B*^*−/−*^ mice spent 20% less time in the light zone (98.7 s. ± 5.2) than the WT controls (123.5 s. ± 5.6, Fig. 4C). Furthermore, there was an interactive effect of age and genotype [*F*(2, 134) = 4.58, *p* = 0.012]. The *Shank3B*^*−/−*^ mice explored the light zone 40% less in adult age (87.6 s. ± 7.3) than their WT controls (145.3 s. ± 12, Fig. 1F). No such result was observed in adolescence (*p* = 0.13) or old age (*p* = 0.63).

### Marble burying

In burying marbles, there was a main effect of age [*F*(2, 134) = 95.47, *p* < 0.001], and the main effect of genotype [*F*(1, 134) = 6.41, *p* < 0.001], but no main effect of sex was observed. Adolescent mice buried almost 30% more marbles (12.6 ± 0.7) than the adult mice (10 ± 1, *p* = 0.039) and roughly 20% more marbles than the old-aged mice (10.4 ± 0.9, *p* = 0.05, Fig. 2B). The *Shank3B*^*−/−*^ mice buried half as many marbles (7.1 ± 0.5) than the WT controls (14.9 ± 0.5, Fig. 2B). No factor interactions were recorded.

## Discussion

Very few studies analyze age as a relevant factor in mouse ASD research and to our knowledge, the present study pushes a step further to elucidate its interplay potential with sex and genetic predisposition in *Shank3B*^*−/−*^ mice. Among the *Shank3B*^*−/−*^ groups, social deficits were observed exclusively in old males. We show that anxiety-like behavior peaks in adult *Shank3B*^*−/−*^ mice. Repetitive grooming and object avoidance are consistently increased in *Shank3*^*−/−*^ mice across their lifespan, and they show reduced locomotor activity and explorative behavior, more profound in females.

Adult mice show the most prevalent social behavior in our study and spend the least time in the respective chambers. While in a chamber, adults prefer interaction over exploration. Previously, 5-month-old WT mice were reported to exhibit abundant social interactions [59], supporting our results. Contrary to our expectations, the *Shank3B*^*−/−*^ mice did not exhibit discernible social deficits in the present study, apart from the old *Shank3B*^*−/−*^ males, which showed nominal social impairment. Social difficulties are one of the most pervasive shortfalls of ASD [13]. The *Shank3*^*−/−*^ mice were produced with deficient sociability [46], yet the only group with a reduced preference for a social partner in our study was the old *Shank3B*^*−/−*^ males. Furthermore, the absolute time they spent in interaction and in chambers indicates they did not engage in much besides the interaction itself. The old *Shank3B*^*−/−*^ males also showed reduced locomotor activity. It is possible that the seeming social deficit could be a result of simply staying near the social partner or the object, rather than actively interacting. Assessing sociability in *Shank3*^*−/−*^ animals produced ambiguous results in the past - no social deficits in *Shank3*^*−/−*^ mice in a large phenotyping study [19] and normal sociability in *Shank3* mutant rats [63] were reported, yet other studies confirm deficient sociability in *Shank3* mutant mice [11, 23]. These inconsistencies could be due to the limited construct validity of SIT. Sociability in rodents comprises mainly direct social interaction [9], a common aspect of other widely used social tasks [60]. SIT only allows the approach of the subject to a social partner, not vice versa, preventing reciprocal interaction. In support of this, phenotyping studies report *Shank3* mutant animals with normal sociability in SIT, but abnormal sociability in other assays [25, 69]. Sociability measured in SIT can thus be laden with insufficient validity, explaining the inability to record wide social deficits in *Shank3B*^*−/−*^ mice in the present study.

Another major ASD hallmark, repetitive behavior [1], was elevated in *Shank3B*^*−/−*^ mice of all age groups in our study. This is in line with many past studies [5, 19, 21, 25, 36, 70], but a recent study reported increased repetitive grooming in adult *Shank3*^*−/−*^ mice, compared to younger *Shank3*^*−/−*^ mice [64]. Noteworthy, the adult *Shank3*^*−/−*^ mice also showed substantial variability in a group of 7 females and only 3 males. Considering we recorded consistent repetitive behavior in *Shank3B*^*−/−*^ mice of all ages with a reasonable sample size (*n* = 21, 20, 26 per respective age group), we believe the results reported by Thabault et al. [64] might be burdened with insufficient sample size and sizable variability. Marble burying is traditionally indicative of rodent repetitive behavior too [65]. However, *Shank3*^*−/−*^ mice exhibit a specific response– an avoidant phenotype toward the marbles [32]. In line with this, *Shank3B*^*−/−*^ mice in our study buried half as many marbles as the WT mice, fully aligning with similar *Shank3*^*−/−*^ phenotyping papers as well [19, 25]. Although not considered a major ASD symptom, children with ASD respond to novelty with avoidance [45]. Adolescent mice buried the most marbles among age groups, which might coincide with a general interest of young mice in exploration, as our adolescent mice exhibited the most rearing too. The interest in exploring outweighs neophobia in young mice, decreasing with age [58]. Consistently, a recent study reported decreased rearing in adult mice, compared to young *Shank3*^*−/−*^ mice [64]. In rodents, rearing is also considered a form of vertical locomotor activity [30]. However, adolescent mice in our study did not differ from the other ages in horizontal locomotor activity, suggesting the increased exploration cannot be attributable to higher overall activity associated with young age. Although up to 95% of ASD patients show restricted interests [31, 66] and about 87% experience some form of motor difficulties [8], but both are not considered core ASD phenotypes [1]. The *Shank3B*^*−/−*^ mice in our study exhibited substantial hypoactivity and reduced exploring, in line with previous findings [19, 25]. Surprisingly, decreased exploration of *Shank3B*^*−/−*^ mice was more evident in females. The majority of animal studies have not described sex differences in rearing [15, 18, 22, 24, 27, 35, 52, 56]. On the other hand, in human patients, males appear to have more restricted interests than females [39]. Importantly, ASD research is historically more focused on male clinical image, and the diagnostic criteria mostly reflect male-specific representations [38]. The discussion on female ASD phenotype has been focused on symptom camouflaging in late years [51, 54]. The reduced exploration we observed in *Shank3B*^*−/−*^ females could shine more light on sex-specific symptomatology of ASD in humans as well.

Anxiety peaks in prevalence during adulthood and gradually decreases into old age, which seems to be associated with age-related brain changes and peripheral physiology [28]. However, mice in our study exhibited the most profound anxiety-like behavior in old age. Some evidence shows increased anxiety-like behavior in old WT mice [58] and it could be hypothesized the changes associated with physiological aging play a role by diminishing connections between the amygdala, insula, and frontal areas, impairing natural fear extinction, and allowing pathological anxiety [3, 4]. Anxiety is further prevalent in youth with ASD [37, 62] and up to 38% of ASD adults are co-diagnosed with some anxiety disorder [43, 67]. We report increased anxiety-like behavior in *Shank3B*^*−/−*^ mice, consistent with previous papers [19, 71]. Another team reported no anxiety-like behavior in 5 to 9-week-old *Shank3*^*−/−*^ mice, replicating the result in a subsequent paper [25, 26]. This corresponds to the adolescence period; the age, however, seems important, since the elevated anxiety-like behavior in *Shank3B*^*−/−*^ mice of our study is the most discernible in adult age. In support, a recent paper reported increasing anxiety-like behavior from adolescence to adulthood, followed by a decline in the old BTBR mouse model of ASD [44].

In conclusion, the presented study is one of the very few to analyze sex-specific ASD-like behavior across the lifespan of *Shank3B*^−/−^ mice. We provide evidence that age affects the core and secondary ASD-like phenotype in mice and needs to be considered. The optimal age period for behavioral testing must be discussed for the research to be truly translational. Our findings are, however, only behavioral and observational in nature, and additional research should extend to biological aging, as well as explore underlying mechanisms by utilizing genetic and pharmacological interventions. Whether the observed age effects are mediated by brain development, changes in sex hormones, or by different, currently unknown factors, needs to be further elucidated.

## Data Availability

The datasets used and/or analyzed during the current study are available from the corresponding author on reasonable request.
